# Children Sustain Their Attention on Spatial Scenes When Planning to Describe Spatial Relations Multimodally in Speech and Gesture

**DOI:** 10.1111/desc.70128

**Published:** 2026-01-20

**Authors:** Ercenur Ünal, Dilay Z. Karadöller, Aslı Özyürek

**Affiliations:** ^1^ Max Planck Institute for Psycholinguistics Nijmegen the Netherlands; ^2^ Middle East Technical University Ankara Turkey; ^3^ Donders Institute for Brain, Cognition and Behaviour Radboud University Nijmegen the Netherlands

**Keywords:** eye‐tracking, gesture, language development, multimodal language, spatial language, visual attention

## Abstract

How do children allocate visual attention to scenes as they prepare to describe them multimodally in speech and co‐speech gesture? In an eye‐tracking study, Turkish‐speaking 8‐year‐old children viewed four‐picture displays depicting the same two objects in different spatial relations as they prepared to describe target pictures depicting left‐right relations. Children's visual attention was sustained on the target picture when they were planning descriptions that expressed the spatial relation multimodally in speech and gesture, but not unimodally in speech only. This pattern persisted regardless of the semantic relation between speech and gesture (i.e., for both complementary gestures that disambiguated speech and redundant gestures that supplemented already unambiguous speech). Importantly, visual attention patterns did not differ across description types while children were previewing the displays before message preparation. These results indicate that multimodal message preparation might place different demands on visual attention than unimodal message preparation, possibly due to the affordances of gestures for expressing spatial relations.

**Summary:**

- We test the idea that the way children attend to spatial relations prior to communication is related to how they end up describing them.
- Children's attention was sustained on spatial scenes when they were planning multimodal spatial descriptions in speech and gesture, but not unimodal descriptions in speech only.
- Planning multimodal descriptions was associated with sustained attention on the spatial scenes regardless of the semantic relation between speech and gesture.

## 1 Introduction

Theories of adult language production propose that speaking begins with conceptualization of the scene to be described, followed by the formulation of a linguistic message, which culminates into an utterance (Levelt 1989). Evidence for the conceptualization part of this model comes from studies of eye‐gaze behavior showing that while or prior to speaking, adults attend to components of the scene that they plan to speak about (Gleitman et al. 2007; Griffin and Bock 2000; Meyer et al. 1998). This idea has also been extended to multimodal language production (Kita and Özyürek 2003), since in natural communication, people use various multimodal signals, including speech and co‐speech gestures, to convey their ideas (Holler and Levinson 2019). According to extended models of multimodal language production, gestures accompanying speech are also planned during message preparation and require conceptualization as does speech planning (Kita and Özyürek 2003). Supporting this view, a recent eye‐tracking study showed that adults attend to scenes differently when they plan to express them multimodally in both speech and gesture as opposed to only in speech (Ünal et al. 2022). Here, we extend the multimodal perspective on language production to children and investigate how children allocate visual attention to scenes as they prepare to describe them multimodally in speech and co‐speech gesture. In doing so, we offer unique insights into processes underlying multimodal message preparation as well as the developmental relation between visual attention and language production.

Children differ from adults in important ways in how they combine gesture with speech to convey meaning (Karadöller, Sümer, & Özyürek, et al. 2024). Adults often produce redundant gestures that express meaning overlapping with speech. Planning these redundant gestures is reflected in their eye‐gaze behavior (Ünal et al. 2022). However, according to models claiming that gesture production does not require conceptualization (de Ruiter 2007; Krauss et al. 2000) such gestures might be dismissed as merely another channel for information already expressed in speech. Thus, a stronger test of the proposal that gestures are also part of conceptualization for message preparation would require cases when gesture expresses complementary information to speech. In fact, children frequently produce complementary gestures that express information that is absent from the accompanying speech (Goldin‐Meadow 1999). Thus, the way children speak and gesture provides an excellent test bed for understanding the demands speech and gesture planning place on visual attention during message preparation.

To investigate this issue, we take the domain of space, specifically how children express left‐right relations between objects. Children younger than 10 do not reliably use locative terms similar to *left* and *right* to express left‐right relations (Abarbanell and Li 2021; Johnston 1984; Karadöller, Sümer, Ünal, et al. 2024). Instead, they typically refer to left‐right relations using general locative terms similar to *side* or *next to*. However, since *side* or *next to* apply to both left and right, they can be ambiguous, especially if both of those spatial relations are present in the context. Nevertheless, children frequently resolve the ambiguity of their spoken descriptions of left‐right relations through gestures (Karadöller, Sümer, Ünal, et al. 2024; Ünal et al. 2025). For example, they complement ambiguous locative terms with spatial gestures to disambiguate the exact spatial relation (e.g., by pointing to one side of the gesture space or placing hands to represent the relative locations of objects). In contrast, adults overwhelmingly refer to left‐right relations using unambiguous locative terms similar to *left* and *right* (Karadöller, Sümer, Ünal, et al. 2024; Ünal et al. 2025). They tend to produce fewer gestures overall, and even when they do so, their gestures tend to occur with *left* and *right* in speech.

These speech and gesture usage patterns present an interesting case for message planning since speech and gesture have different affordances for expressing spatial relations. Speech relies on categorical forms which refer to perceptually discriminable exemplars of a given spatial relation with the same locative term. Gestures are articulated in three‐dimensional space, and how they represent objects and their locations maps onto the way objects are located in real space in an analogue way. Because of this space‐to‐space mapping, gestures can express visual‐spatial information not found in the accompanying speech. For example, gestures may represent the properties of the objects (e.g., shape or size) or the spatial relation itself (e.g., orientation of or distance between the objects). This is true for gestures that have different semantic relations with the accompanying speech—that is, both complementary gestures disambiguating speech and redundant gestures accompanying unambiguous speech. Thus, multimodal descriptions of spatial relations can express more visual‐spatial information than unimodal descriptions of the same spatial relations. If gestures are part of conceptualization for message preparation, these affordances of multimodal spatial expressions should engage visual attention differently than unimodal spatial expressions in speech alone.

Beyond its significance for multimodal message preparation, this question informs developmental accounts of the relation between visual attention and language production. Few studies to date have combined eye‐gaze based measures of visual attention with spoken language production in children, and this work revealed conflicting findings. Some studies have reported that preschoolers show the same visual attention patterns that adults do during message preparation (Bunger et al. 2016, 2021). Other studies revealed that even though children attend to core components of scenes, in contrast to adults, they do not always mention these components in their speech (Bunger et al. 2012; Davies and Kreysa 2017, 2018). However, this work might underestimate the link between eye‐gaze behavior and language production in children due to its focus on how children describe scenes in speech. This is especially a concern for developmental evidence given that children are much more likely than adults to complement speech with gestures. If multimodality of descriptions plays a key role in how children glean information from the visual world during message preparation, we may need to reconsider previous developmental evidence on the relation between visual attention and language production.

### 1.1 Present Study

Here, we ask if children allocate visual attention differently when planning to describe spatial relations multimodally compared to unimodally; and regardless of the type of semantic relation between speech and gesture. To address these questions, we elicited spatial descriptions from children while their eye‐movements were recorded. Participants viewed a set of four pictures, each depicting the same two objects, but in a different spatial relation. One picture was designated as the target that they had to describe to an addressee. The target always depicted a left or right relation. Children's descriptions were either unimodal (*left/right* or *side* only) or multimodal (*left/right* or *side* with gesture).

We conducted three comparisons of eye‐gaze behavior while planning multimodal descriptions versus a unimodal baseline. Each comparison tested a different type of multimodality. The comparison between *left*/*right* with gesture to *left*/*right* only descriptions tested whether visual attention differs between multimodal descriptions with redundant gestures versus unimodal descriptions with similar speech alone. This would replicate previous findings from adults (Ünal et al. 2022). The next two comparisons involved cases in which gesture expressed complementary information to speech. Thus, the second comparison was between *side* with gesture and *left/right* only descriptions. Both descriptions disambiguate the spatial relation—but the first does so multimodally by combining speech with gesture, and the second unimodally through speech only. Finally, the comparison between *side* with gesture to *side* only descriptions tested whether visual attention differs between multimodal descriptions with complementary gestures that disambiguated speech than unimodal descriptions with similar speech that does not disambiguate the spatial relation. If the affordances of gestures for expressing visual‐spatial information engages visual attention differently, there should be more attention allocated to the target picture when planning multimodal spatial descriptions compared to unimodal spatial descriptions in all three cases.

## 2 Method

The methods reported in this study were approved by the Humanities Ethics Assessment Committee of Radboud University and the Ministry of Education in Istanbul, Turkey.

### 2.1 Participants

Data were collected from 24 children^1^ who were native speakers of Turkish (14 females, mean age = 8;6; range = 6;7–9;5). All had learned Turkish from birth and as their first language, and were not proficient in another language. Participants were tested in Istanbul, Turkey. The sample was recruited from a public elementary school in an urban environment serving families with a middle socioeconomic background. Data from three additional children were excluded due to being bilingual (*n* = 1) or failing to describe more than a third of the items (*n* = 2). All exclusions were made prior to data analyses. Participation was voluntary and compensated with a small gift.

### 2.2 Stimuli

**FIGURE 1:**
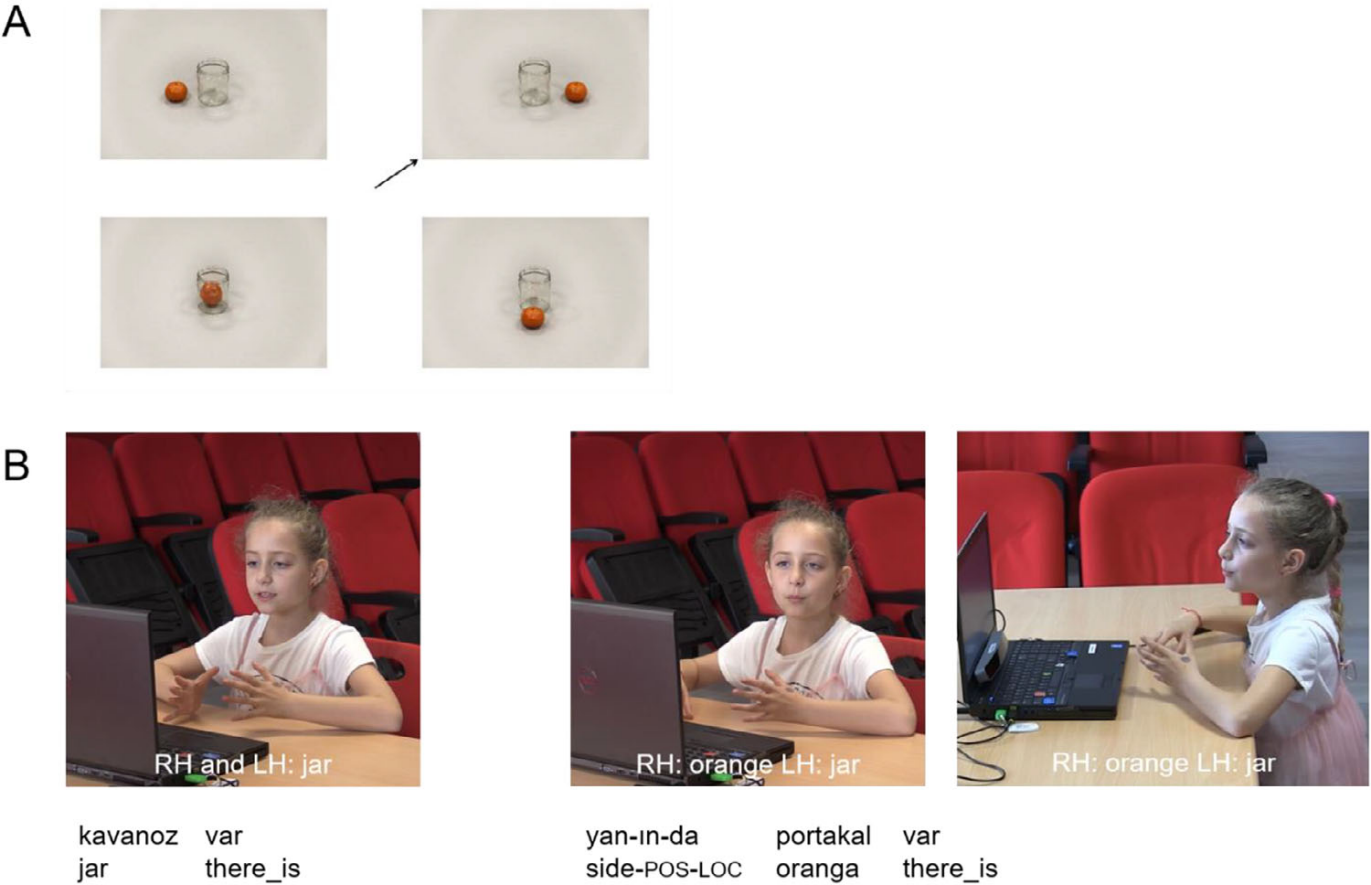
(A) Stimuli in critical trials (B) Example of spatial expressions in speech and gesture. (A) The target picture (indicated by the arrow) depicted a left or right relation. One of the non‐target pictures always depicted the contrastive spatial relation. The other two non‐target pictures (bottom pictures) depicted topological (in or on) and lateral (front or behind) configurations. (B) The speech that the gesture overlaps with is presented under the pictures. The second gesture is presented from both front and side angles.

Stimuli were 84 displays depicting four pictures (Figure 1A). Each picture depicted the same two objects in a different spatial relation. Ground objects served as the reference object. They were placed in the center of the picture and remained in the same location in all four pictures. They did not have any intrinsic sides that could be determined by their shape. Figure objects were the object to be located. They were placed in different locations with respect to the ground object in each of the four pictures. The only distinguishing feature of individual pictures within a display was the spatial relation between the objects. In each display, one picture was designated as the target picture that the participants had to describe to an addressee. In critical trials (*n* = 14), the target always depicted a left or right configuration, and one of the non‐targets depicted the contrastive configuration. The remaining displays were fillers. Each figure object was presented only once. Each ground object was presented four times, but each time together with a different figure object. Each participant saw the displays in a different pseudorandomized order with the following constraints: the same ground object or the same target spatial relation did not appear in two or more consecutive trials. Within each display, the assignment of individual pictures to a location on the screen was randomized.

### 2.3 Procedure

Children were seated 60 cm away from a Dell Precision M4800 laptop in a quiet room at their school. Stimuli were presented with Presentation software, and eye gaze was recorded using an SMI RED 250 eye‐tracker at a sampling rate of 250 Hz. After a 5‐point calibration and validation, children completed 84 trials. Each trial consisted of a fixation screen (2000 ms), followed by the four‐picture display (1000 ms) and an arrow pointing to the target picture (500 ms). Finally, the four‐picture display remained on the screen for an additional 2000 ms until a visual mask was presented. Participants described the target picture during the visual mask. Eye movements were not recorded during the visual mask. After the participants finished describing the target picture, they pressed enter to initiate the next trial.

At the beginning of the experiment, participants completed three practice trials with displays similar to the ones used in the actual experiment to familiarize themselves with the task. During the practice trials, if participants failed to follow the instructions, the experimenter repeated them. To maximize the informational needs, there was a confederate addressee who had to use the participant's description to find the target picture among the same four pictures on their tablet. Children knew that the addressee also had the same four pictures, but arranged in a different way and without the arrow. The addressee did not provide any feedback on whether the description was correct. When participants did not mention the spatial relation, the addressee asked for the location of the figure (''Where is [Figure object]?) only once. To ensure that gestures were produced spontaneously, gesture use was not mentioned in the instructions. Sessions were recorded with two Canon video cameras from front and side angles for later coding. The task lasted approximately 20 min.

### 2.4 Coding

Participants’ descriptions were transcribed and coded for spatial expressions in speech and gesture by native speakers of Turkish using ELAN software (Lausberg and Sloetjes 2009). Data from 16 trials (0.5% of the data) in which participants only labeled the objects in speech and did not mention the spatial relation between them were excluded. When participants expressed the spatial relation in speech, they either used specific spatial terms corresponding to *left or right* or general spatial terms corresponding to *side* or *next to*.

For gesture, we segmented gesture strokes (the most meaningful part of the hand movement) that accompanied speech and represented the location of the figure and/or ground object. We did not code gestures that only depicted the shape of the objects without locating them, or non‐representational gestures that did not convey any meaning. A spatial gesture was coded as present if participants represented the relative location of the figure and/or ground objects using directional pointing gestures or by placing their hands in the gesture space in relation to each other (Figure 1B). The interrater reliability for coding gesture presence was calculated on 25% of the gesture data. This resulted in a raw agreement of 83.7% and kappa = 0.671. indicating substantial agreement.

Based on spatial encoding in speech and gesture descriptions were categorized into four types: *side* only (20.6% of the descriptions), *side* + gesture (46.8% of the descriptions), *left/right* only (12.2% of the descriptions), and *left/right* + gesture (20.3% of the descriptions).

### 2.5 Data Processing

In each display, five non‐overlapping Areas of Interest (AOIs) were defined (one for each picture and one for the arrow) using SMI BeGaze software. We computed whether a fixation fell into one of the AOIs in each successive 50 ms time bin from the onset of the preview display until the visual mask display (0–3500 ms). Trials with greater than 50% trackloss were excluded from the analysis (0.3% of the data). No participants were excluded based on the apriori criterion of having more than 45% trackloss across all trials. All exclusions were made prior to the analyses.

## 3 Results

**FIGURE 2:**
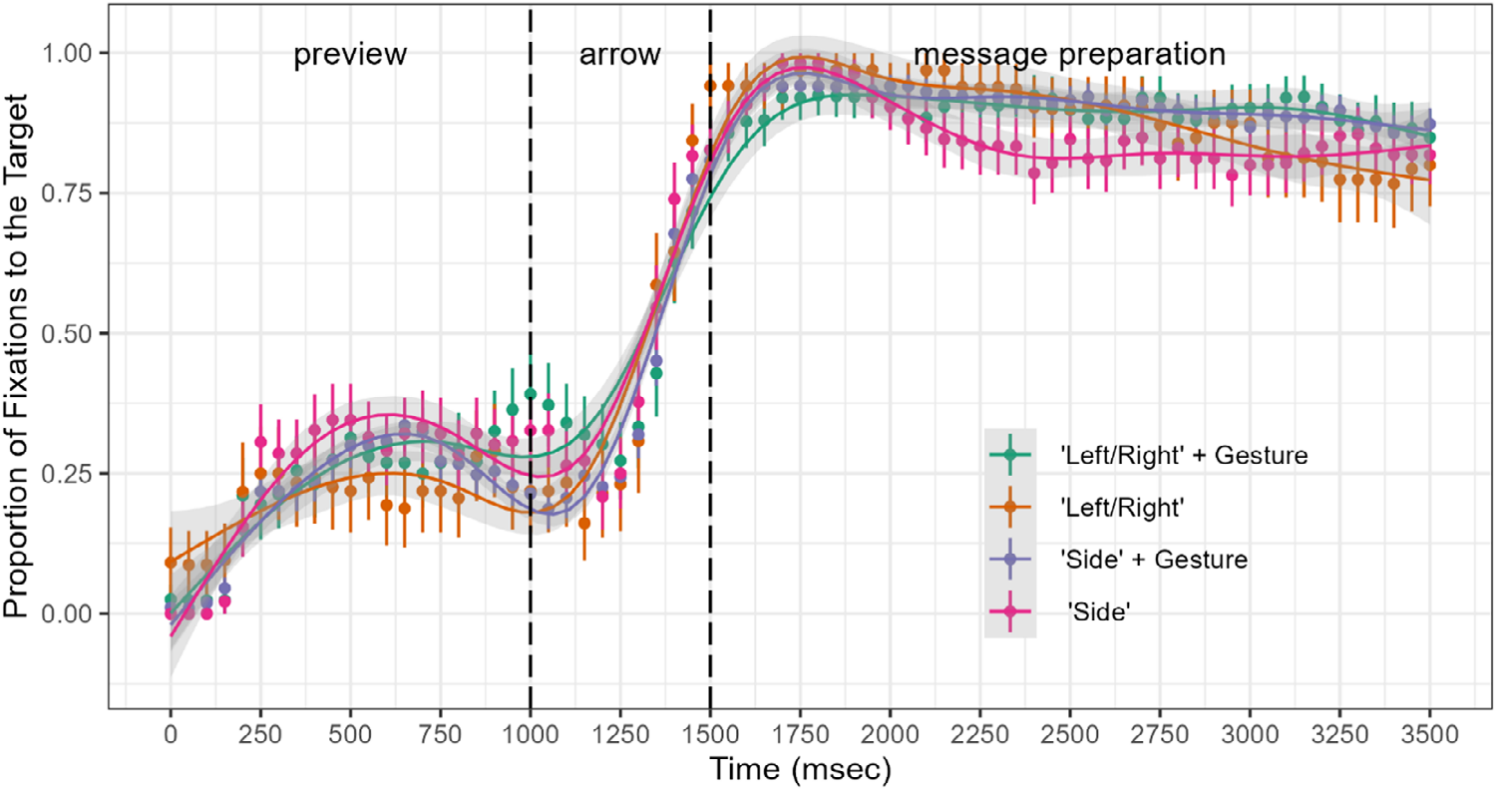
Looks at the target picture across description types.

The time course data of eye movements were analyzed in successive 50 ms time bins in two windows: the preview window (0–1000 ms) and the message preparation window (1500–3500 ms). The visual displays in the two windows were identical. However, in the preview window, participants did not yet know which picture they had to describe. Thus, the preview window served as a baseline to ensure that viewing patterns did not systematically differ prior to message preparation. Figure 2 shows the proportion of fixations to the target picture over time across description types.

We tested whether the time course of the attention allocated to the target picture changed across description types using binomial logistic regressions. The dependent measure was binary values for whether or not the target picture was fixated in each time bin. Subjects and Items were added as random intercepts. Time (scaled and centered), description type (*left/right* + gesture, *left/right* only, *side* + gesture, *side* only), and interaction between the two were added as fixed factors. The fixed effect of description type was tested with planned contrasts using forward difference coding, where each contrast compares one level of the fixed effect to the next adjacent level. Parameter estimates from the models are presented in Table 1. All models were fit with *glmer* function of *lme4* package (Bates et al. 2015) in *R* (R Core Team 2024). We corrected for multiple comparisons using the False Discovery Rate with the *p.adjust* function in *R*. Data and analysis code are available at https://osf.io/2zmxt/.

**TABLE 1:** Parameter estimates of the fixed effects of description type and time on the looks to the target in the preview and message preparation windows.

	*β*	SE	*z*	*p* value
**Preview window**				
Intercept	−1.193	0.143	−8.314	<0.001
Description _[_ *_left_*_/_ *_right_*_+ gesture vs._ *_left_*_/_ *_right_*_]_	0.291	0.180	1.616	0.106
Description _[_ *_left_*_/_ *_right_*_vs._ *_side_*_+ gesture]_	−0.332	0.184	−1.803	0.071
Description _[_ *_side_*_+ gesture vs._ *_side_*_]_	−0.396	0.109	−3.637	0.001
Time	0.176	0.041	4.273	<0.001
Description _[_ *_left_*_/_ *_right_*_+ gesture vs._ *_left_*_/_ *_right_*_]_ * Time	0.214	0.137	1.561	0.119
Description _[_ *_left_*_/_ *_right_*_vs._ *_side_*_+ gesture]_ * Time	−0.047	0.120	−0.391	0.696
Description _[_ *_side_*_+ gesture vs._ *_side_*_]_ * Time	−×0.037	0.090	−0.408	0.683
**Message preparation window**				
Intercept	2.591	0.308	8.401	<0.001
Description _[_ *_left_*_/_ *_right_*_+ gesture vs._ *_left_*_/_ *_right_*_]_	−0.212	0.180	−1.178	0.239
Description _[_ *_left_*_/_ *_right_*_vs._ *_side_*_+ gesture]_	−0.564	0.217	−2.600	0.019
Description _[_ *_side_*_+ gesture vs._ *_side_*_]_	−0.120	0.107	−1.127	0.260
Time	−0.392	0.040	−9.689	<0.001
Description _[_ *_left_*_/_ *_right_*_+ gesture vs._ *_left_*_/_ *_right_*_]_ * Time	0.906	0.137	6.608	<0.001
Description _[_ *_left_*_/_ *_right_*_vs._ *_side_*_+ gesture]_ * Time	−0.752	0.124	−6.081	<0.001
Description _[_ *_side_*_+ gesture vs._ *_side_*_]_ * Time	0.226	0.086	2.638	0.017

*Note*: Formula in *R* for each window: glmer(target ∼ DescriptionType*scaled_bin + (1|pp) + (1|stim)). The fixed effect of description type was tested with forward difference coding, in which each level is compared with the subsequent level. Because the reference level shifts across contrasts, the effects that go in the same direction may have coefficients in different directions. Contrasts 1 and 3 have multimodal description types as reference levels (*left*/*right* + gesture and *side* + gesture, respectively) and Contrast 2 has a unimodal description type as reference level (*left*/*right* only). Accordingly, a negative coefficient for Contrast 2 indicates an effect in the same direction as positive coefficients for Contrasts 1 and 3.

In the preview window, looks to target picture increased over time as indicated by a significant positive slope for the term for time. Crucially, the rate of increase in target looks did not change across description types as the time did not interact with any of the contrasts testing the effect of description type (Table 1). Thus, regardless of how they ended up describing the spatial relation in the target, children viewed it similarly during the preview window—that is, before they learned which picture they would be describing.^2^

In the message preparation window, looks to target picture decreased over time as indicated by a significant negative slope for the term for time. However—and unlike in the preview window—the time course of target looks varied across description types as time had a significant interaction with all of the contrasts testing the effect of description type (Table 1). Compared to *left/right* only descriptions, planning *left/right* + gesture descriptions changed the negative trend in time in the opposite direction: the decrease in target looks was reversed for *left/right* + gesture descriptions. The same patterns characterized the difference between *side* only versus *side* + gesture descriptions. Finally, the decrease in target looks was faster over time when planning *left/right* only compared to *side* + gesture descriptions. These findings indicate that visual attention was sustained on the target picture when planning multimodal descriptions but not unimodal descriptions of left‐right relations.

In order to further evaluate variations in visual attention patterns in relation to planning different types of descriptions, we conducted three sets of exploratory analyses. First, to further test that multimodal message preparation is associated with sustained visual attention regardless of the semantic relation between speech and gesture, we explored eye‐gaze patterns during message preparation for *left‐right* + gesture and *side* + gesture descriptions. We reasoned that if visual patterns are due to the multimodality of the descriptions, then the time course of visual attention should not differ across *left‐right* + gesture descriptions in which gesture is redundant and *side* + gesture descriptions in which gesture is complementary to speech. Supporting this possibility, the time course of target looks did not differ across these two types of multimodal descriptions (*β* = 0.154, SE = 0.912, *z* = 1.689, *p* = 0.183).

Next, we evaluated whether visual attention patterns associated with multimodal message preparation generalizes beyond left‐right relations. We conducted an exploratory analysis on a subset of the filler trials that had front‐behind relations as the target. This is because the descriptions of front‐behind relations consisted of the same multimodal and unimodal description types as for the critical left‐right relations, though the distribution of the description types was different. We replicated all the effects from the analyses of the critical left‐right relations. Visual attention was sustained on the target when children were planning multimodal descriptions but not unimodal descriptions of front‐behind relations, suggesting that multimodal message preparation is consistently associated with sustained attention on the spatial scene (see Supporting Information for a detailed description and model results).

Finally, although the goal of our study was to test whether the time course of visual attention changes across multimodal and unimodal descriptions, we also explored eye‐gaze patterns during message preparation for *left‐right* only and *side* only descriptions to connect our findings to previous work on the relation between children's speech production and visual attention. As expected, children allocated less attention to the target picture over time while planning *side* only descriptions that did not disambiguate the spatial relation compared to *left/right* only descriptions that disambiguated the spatial relation (*β* = −0.526, SE = 0.133, *z* = −3.961, *p* < 0.001). These findings cohere with previous findings showing that eye‐gaze patterns in message preparation reflect variations in the content of descriptions in speech.

## 4 Discussion

Conceptualization is proposed to be the first step of planning a description, providing input for the later stages of processing, including the content and form of the description (Levelt 1989; cf. Kita and Özyürek 2003). The relation between conceptualization and language production is well‐established in adults for both speech (Gleitman et al. 2007; Meyer et al. 1998) and multimodal language in speech and co‐speech gesture (Ünal et al. 2022). Our goal here was to seek evidence from eye‐gaze behavior for the link between conceptualization and multimodal message preparation in children and test whether this link persists regardless of the semantic relation between speech and gesture.

Our key prediction was that planning to express spatial relations multimodally in speech and gesture would engage visual attention differently than doing so unimodally in speech alone, due to the affordances of gestures for expressing spatial relations. Supporting this, we first showed that children allocated more attention to visual‐spatial information while they were planning to produce redundant gestures alongside speech (*left/right* + gesture), compared to similar spoken descriptions without any gesture (*left/right* only). This replicated earlier findings with adults (Ünal et al. 2022). In a novel contribution, we further demonstrated that the time course of visual attention during message preparation changed when planning gestures expressing complementary information to speech. Specifically, visual attention was sustained on the target spatial relation while children were planning to express the spatial relation multimodally by complementing speech with gesture (*side* + gesture). By contrast, children allocated less attention to the spatial relation over time while planning to express the same information unimodally in speech only (*left*/*right*). Furthermore, children allocated attention to visual‐spatial information differently when planning descriptions with complementary gestures that disambiguated speech (*side* + gesture), compared to when they planned ambiguous spoken descriptions without any gesture (*side* only).

Together, these findings provide strong evidence for the proposal that gesture requires conceptualization as does speech planning (Kita and Özyürek 2003) by showing differences in eye‐gaze behavior related to planning multimodal descriptions of spatial relations. Importantly, planning multimodal spatial descriptions engages visual attention differently and places additional demands on attention to visual‐spatial information for both gestures that were redundant with respect to the spatial relation and those that complemented speech by expressing spatial information absent from the accompanying speech. This strongly suggest that the changes in the time course of visual attention are likely due to the space‐to‐space mapping in spatial expressions in gestures, which can express additional visual‐spatial features of the objects and the spatial relations.

By uncovering previously hidden links between visual attention and language production in children, our findings offer novel insights for developmental accounts of language production. Recall that existing evidence on the link between eye‐gaze behavior and the content of children's (spoken) descriptions is mixed. Our findings call for a reevaluation of both types of prior evidence. One line of work showed that children are broadly similar to adults and show tight relations between attention allocation and message preparation (Bunger et al. 2016, 2012, 2021). Importantly, this work also showed similar visual attention patterns during message preparation as long as the content of spoken descriptions were similar. Our findings highlight the importance of going beyond the content of expressions and considering the multimodal nature of language for uncovering the subtleties in the dynamics between visual attention and language production. Another line of work found mismatches between viewing and speaking behavior in children (Davies and Kreysa 2017, 2018); such that children often did not speak about the details that they had attended to. By showing that eye‐gaze patterns also reflect children's descriptions in gesture, we offer an alternative explanation for this previous evidence, namely that the detailed omitted from speech might have been expressed in complementary gestures.

The present work has some limitations that open directions for future work. First, our findings show that children who ended up describing spatial relations differently had previously attended to them differently while planning those descriptions; but do not establish that the relation between visual attention patterns and description types was causal. Future work can address this by manipulating the multimodality of descriptions as well as the semantic relation between speech and gesture. Such work can also benefit from using specific time windows for the analysis of eye‐gaze patterns (e.g., from preview to conceptualization to description onset) to precisely identify when eye‐gaze patterns diverge from each other across description types. Finally, the present study used looks to the target picture as an indicator of children's attention to spatial information, as all four pictures included the same two objects, and the spatial relation between them was the distinguishing feature. Alternatively, one might evaluate the attention allocated Figure and Ground objects separately or measure the transitions of fixations between them. Although technically not feasible in the current study, this approach might be valuable for future work, especially for designs relying on descriptions of single pictures depicting a spatial relation.

All in all, our findings highlight that a multimodal approach to language provides not only a more precise estimate of children's communicative abilities but also a better characterization of the cognitive processes underlying the production of these communicative signals.

## Funding

This research was supported by NWO‐VICI grant 277‐70‐013 awarded by The Dutch Research Council to A.Ö.

## Ethics Statement

The methods reported in this study were approved by the Humanities Ethics Assessment Committee of Radboud University and the Ministry of Education in Istanbul, Turkey.

## Conflicts of Interest

The authors declare no conflicts of interest.

## Supporting information




**Supporting File 1**: desc70128‐sup‐0001‐SupMat.docx

## Data Availability

The data and the code that support the findings of this study are available in Open Science Framework https://osf.io/2zmxt/.

## Endnotes

1 Sample size was determined based on the following constraints. Data reported in this article were collected as part of a larger project that also involves comparisons between deaf children acquiring Turkish Sign Language and hearing children acquiring spoken Turkish. For deaf participants, we collected data from all children attending the deaf schools in Istanbul, Turkey, whose parents provided consent and met our selection criteria. The number of hearing children was matched to the number of deaf signing peers. The sample size is comparable to recent developmental work combining eye‐tracking measures with language production (Bunger et al. 2016, 2021; Davies and Kreysa 2017, 2018).

2 Although there was a significant effect of description type for side + gesture versus *side* only comparison in the preview window, this is unlikely to indicate a systematic difference in viewing patterns that would result in the differences in the message preparation window. First, this difference did not interact with the term for time, which was the critical effect for testing how the allocation of visual attention changes over time. Second, this effect is in the opposite direction than the same comparison in the message preparation window—*side* only descriptions were associated with more target looks than *side* + gesture descriptions. Thus, even if there was a difference in viewing patterns, knowing which picture to describe and planning to describe it in speech and gesture seems to have reversed this pattern.
