# Supplementary material for: Children Sustain Their Attention on Spatial Scenes When Planning to Describe Spatial Relations Multimodally in Speech and Gesture
Source: Dev Sci. 2026 Jan 20;29(2):e70128. doi: 10.1111/desc.70128 (PMC12817325; doi:10.1111/desc.70128)
Supplement: Supplementary file 1 — Supporting File 1: desc70128‐sup‐0001‐SupMat.docx [file DESC-29-e70128-s001.docx]

**Supplementary Material**

**Analysis of Filler Trials**

In filler trials, the target image depicted a front, behind, in, or on relation. Of those relations, front-behind were similar to the critical left-right relations in that they were also axial relations that involved a viewpoint. Further, children in this age range describe front-behind relations using both specific spatial terms corresponding to *front or behind* or general spatial terms corresponding to *side* or *next to.* Children often gestured together with these spoken descriptions. Thus, the types of descriptions produced for these relations are quite similar to the descriptions in critical trials. However, in and on relations are topological, do not involve a viewpoint and were almost always described with specific spatial terms corresponding to *in* or *on* (99.4 % of the descriptions in our data). These descriptions are often not accompanied with gesture. For that reason, the analysis of the filler data was performed on front-behind relations.

The description data for front and behind trials had the following distribution: *side* only (8.1% of the descriptions), *side* + gesture (21.7% of the descriptions), *front/behind* only (38.3% of the descriptions), and front*/behind* + gesture (31.8% of the descriptions). Fig.SM1 shows the proportion of fixations to the target picture over time across description types in filler trials.

**Figure SM1**

*Looks to the target picture across description types for filler trials*


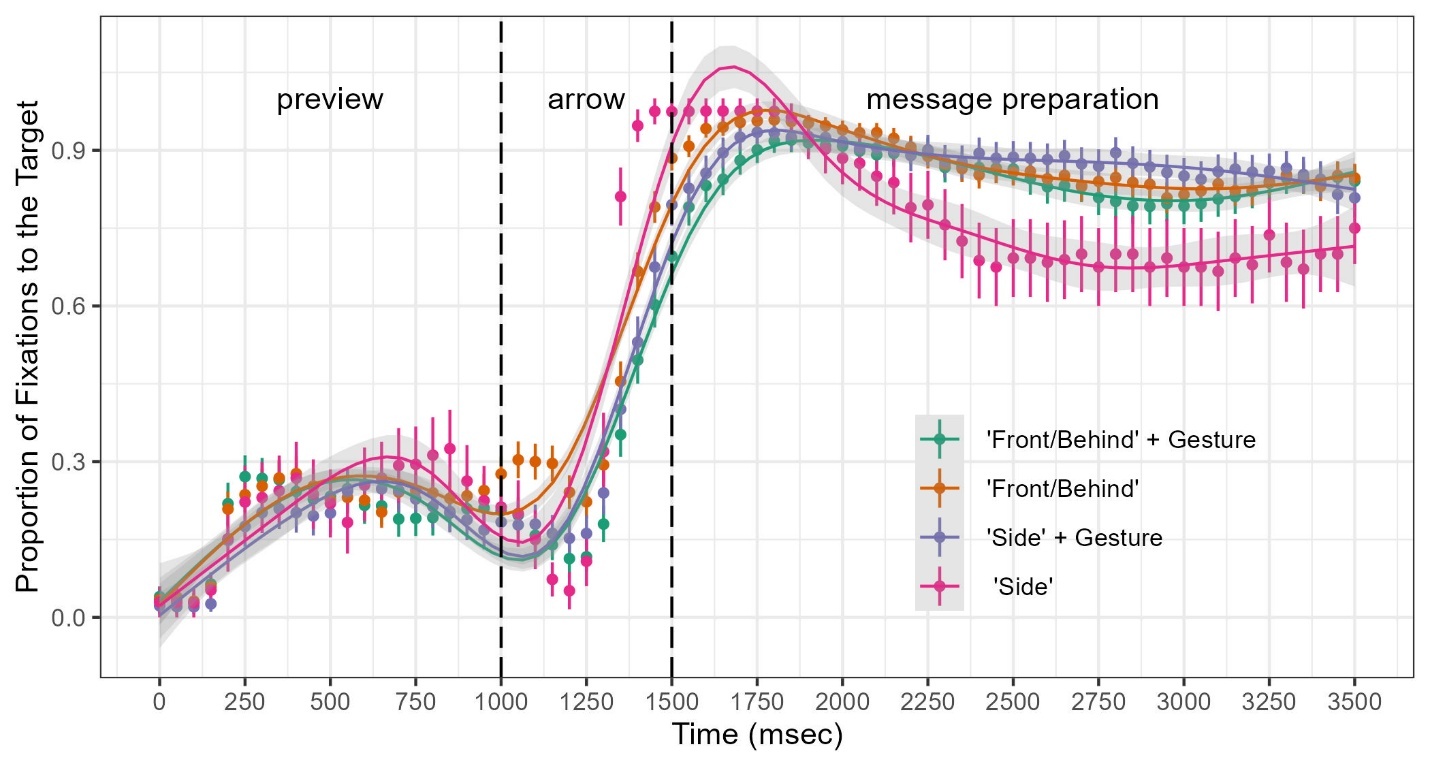


The models were fit on the data from filler trials using the same strategy as in the data from critical trials described in the manuscript. **In the preview window,** the time course of looks to target picture did not vary across description types, since the time term did not interact with any of the contrasts testing the effect of description type. However, **in the message preparation window**, the time course of target looks varied across description types as time had a significant interaction with all of the contrasts testing the effect of description type. All three contrasts revealed that visual attention was sustained on the target picture when they planning multimodal descriptions but not when planning unimodal descriptions (*front/behind* + gesture versus *front/behind* only descriptions, *side* + gesture vs. *front/behind* only descriptions, and *side* + gesture versus *side* only descriptions (Table SM1). These patterns for front and behind trials mirror the patterns for critical left and right trials.

**Table SM1**

*Parameter estimates of the fixed effects of description type and time on the looks to the target in preview and message preparation windows for filler trials*

|  |  | **β** | **SE** | **z** | **p value** |
| --- | --- | --- | --- | --- | --- |
| **Preview window** | | | | | |
|  | Intercept | -1.265 | 0.148 | -8.554 | <.001 |
|  | Description _[_*_front/behind_* _+ gesture vs._ *_front/behind_*_]_ | -0.462 | 0.088 | -5.237 | <.001 |
|  | Description _[_*_front/behind_* _vs._ *_side_* _+ gesture]_ | 0.502 | 0.133 | 3.780 | <.001 |
|  | Description _[_*_side_* _+ gesture vs._ *_side_*_]_ | -0.517 | 0.146 | -3.541 | <.001 |
|  | Time | 0.100 | 0.032 | 3.118 | .002 |
|  | Description _[_*_front/behind_* _+ gesture vs._ *_front/behind_*_]_ * Time | -0.119 | 0.068 | -1.744 | .081 |
|  | Description _[_*_front/behind_* _vs._ *_side_* _+ gesture]_ * Time | -0.054 | 0.075 | -0.724 | .469 |
|  | Description _[_*_side_* _+ gesture vs._ *_side_*_]_ * Time | -0.077 | 0.109 | -0.703 | .482 |
| **Message preparation window** | | | | | |
|  | Intercept | 2.298 | 0.233 | 9.868 | <.001 |
|  | Description _[_*_front/behind_* _+ gesture vs._ *_front/behind_*_]_ | -0.149 | 0.072 | -2.070 | .038 |
|  | Description _[_*_front/behind_* _vs._ *_side_* _+ gesture]_ | 0.000 | 0.107 | 0.004 | .997 |
|  | Description _[_*_side_* _+ gesture vs._ *_side_*_]_ | 0.090 | 0.116 | 0.782 | .434 |
|  | Time | -0.397 | 0.026 | -15.022 | <.001 |
|  | Description _[_*_front/behind_* _+ gesture vs._ *_front/behind_*_]_ * Time | 0.277 | 0.057 | 4.839 | <.001 |
|  | Description _[_*_front/behind_* _vs._ *_side_* _+ gesture]_ * Time | -0.234 | 0.063 | -3.711 | <.001 |
|  | Description _[_*_side_* _+ gesture vs._ *_side_*_]_ * Time | 0.570 | 0.088 | 6.451 | <.001 |

*Note.* Formula in *R:* glmer(target ~ DescriptionType*scaled_bin + (1|pp) + (1|stim))

The fixed effect of description type was tested with forward difference coding, in which each level is compared with the subsequent level. Because the reference level shifts across contrasts, the effects that go in the same direction may have coefficients in different directions. Contrasts 1 and 3 have multimodal description types as reference levels (*front/behind* + gesture and *side* + gesture, respectively) and Contrast 2 has a unimodal description type as reference level (*front/behind* only). Accordingly, a negative coefficient for contrast 2 indicates an effect in the same direction as positive coefficients for contrasts 1 and 3.
